# Construction of a co-expression network and mining of core genes for genic male sterility in sweet pepper based on transcriptome and WGCNA analysis

**DOI:** 10.3389/fpls.2025.1668660

**Published:** 2025-10-21

**Authors:** Yaning Meng, Zhe Zhang, Xinxin Li, Hongxiao Zhang, Zhanghong Yu, Yanqin Fan, Libin Yan

**Affiliations:** ^1^ Institute of Cash Crops, Hebei Academy of Agriculture and Forestry Sciences, Shijiazhuang, China; ^2^ Hebei Normal University of Science & Technology, Qinhuangdao, China; ^3^ Hebei Province Engineering Research Center for Vegetables, Shijiazhuang, China

**Keywords:** sweet pepper, genic male sterility, transcriptome, weighted gene co-expressionnetwork analysis (WGCNA), core genes

## Abstract

**Background:**

This study investigates the molecular mechanisms underlying genic male sterility in sweet pepper by examining the correlation between differentially expressed genes during various stages of anther development and pollen sterility.

**Results:**

We collected anthers from both pure fertile and sterile plants of the F2 generation of the sweet pepper genic male sterility line AB91 at three distinct developmental stages for comprehensive transcriptome sequencing. By integrating KEGG enrichment analysis with weighted gene co-expression network analysis (WGCNA), we identified Starch and sucrose metabolism (ko00500) and Phenylpropanoid biosynthesis (ko00940) as key metabolic pathways regulating genic male sterility in sweet pepper AB91. Additionally, we uncovered two specific gene modules (blue and brown) associated with genic male sterility. A total of 320 genes, which exhibited significant differences (MM≥0.8) between these two modules, were found to be highly expressed after the formation of tetrads. This finding suggests that the period following tetrad formation is a critical juncture for changes in gene expression related to genic male sterility in sweet pepper AB91. By comparing these genes with their homologs, we identified six differentially expressed genes that can serve as core genes related to genic male sterility and play essential roles during microspore development.

**Conclusions:**

This research provides a theoretical foundation for elucidating the molecular mechanisms of genic male sterility in sweet pepper and lays the groundwork for expanding fertility gene resources and advancing hybrid breeding efforts in this important crop.

## Introduction

Sweet pepper, *Capsicum annuum* var.*grossum*, a member of the Solanaceae family and *Capsicum genus*, is an annual or perennial crop and a globally significant vegetable. The utilization of hybrid vigor in sweet pepper can increase yields by 30% to 50% compared to conventional varieties (Mark and Peter, 2010). Hybrid breeding using male sterile mutants can eliminate the need for manual emasculation, reducing the cost and difficulty of seed production and significantly enhancing seed purity (Dhaliwal and Jindal, 2014). The production of F1 hybrid seeds using male sterile lines is a crucial pathway for hybrid vigor utilization (Kim and Zhang, 2018). Therefore, research on male sterility has been highly valued by scholars worldwide (Su et al., 2016).

We identified a natural male sterile source in the Ingber pepper population and developed a proprietary pepper genic male sterile line AB91 through hybridization, selfing, and test crossing (Yanqin and Jingyin, 1993). The sterility trait is stably inherited and controlled by a pair of recessive genic sterility genes, with complete sterility, stable infertility, no adverse cytoplasmic effects, a wide range of restorer sources, and great freedom in crossing, making it easy to obtain strong heterotic combinations. To date, 16 hybrid pepper varieties developed using AB91 have been nationally and provincially approved and are widely used in production, addressing the problem of manual emasculation in F1 hybrid pepper seed production in China.

The growth and development of plant floral organs are regulated by the coordinated expression of numerous nuclear genes. Abnormalities in the normal expression and transcription of certain nuclear genes may lead to disruptions in energy and material metabolism within the plant, changes in hormone levels, and ultimately, the occurrence of male sterility. During the development of male sterile lines, genes such as *MYB*, *PHD*, *TDR*, *AMS*, *bHLH*, *WRKY*, and *TDF* exhibit differential expression (Sheng et al., 2017; Schuber et al., 2019; Wan et al., 2019; Lu et al., 2020; Pan et al., 2020). Transcription factors *bHLH*, *DYT1*, *TDF1*, and *AMS* act early in tapetal development, while *MYB* and PHD-figure proteins are subsequently activated to regulate late tapetal development and pollen wall formation (Zhu et al., 2011). In rice, *DYT* plays a crucial role in maintaining tapetal development during early meiosis, and *TDR* interacts with *bHLH* to regulate tapetal development and degeneration by modulating protease expression (Ko et al., 2014). In Arabidopsis, *WRKY34* and *WRKY2* can interact with VQ20 protein in the nucleus to form a complex. VQ20 may regulate pollen development and function by affecting the transcriptional functions of *WRKY34* and *WRKY2* (Lei et al., 2017). Zhang et al (Zhang et al., 2021)identified a specific gene, *CLATM1*, in nuclear male sterile watermelon plants using map-based cloning. This gene encodes a bHLH transcription factor missing 10 bp and can activate its own transcription by binding to the promoter. Chen et al (Chen and Liu, 2014). found through expression and functional analysis of differential proteins and genes in rice sterile plants that heat shock proteins (Hsps) in anthers are involved in protein folding. As rice plants develop, the expression of Hsps in sterile plants increases, leading to changes in protein folding. Additionally, *GH16*, a plant hormone signal transduction gene, is involved in anther cell wall development, and the cuticle, a significant component of the anther cell wall, participates in the biosynthesis of phenolic compounds and fatty acids. In phenolic compound biosynthesis, *PAL* is a key upstream gene and is lowly expressed in sterile plants. In fatty acid biosynthesis, the expression of 3-hydroxyacyl-CoA dehydrogenase is lower in sterile plants than in fertile plants. During this process, *HOTHEAD* precursors are highly expressed in sterile plants, which may lead to pollen abortion in WXS(S) (Wu et al., 2012). Moreover, metabolic pathways and starch and sucrose metabolism are involved in the development and energy supply of WXS(S). Although the molecular mechanisms of pollen development in other genic sterile plants have been studied, the mechanisms underlying male sterility in sweet pepper genic male sterile line AB91 are not well understood, and the regulatory mechanisms of sterility are not yet clear.

In this study, we used the sweet pepper genic male sterile line AB91 to investigate the key genes and regulatory pathways leading to AB91 genic male sterility at different stages of microspore development, clarify the regulatory mechanisms of genic sterility genes causing another abortion, and provide theoretical and technical support for expanding pepper nuclear sterility gene resources and promoting molecular breeding in pepper.

## Materials and methods

### Plant materials and treatments

The sweet pepper recessive genic male sterile line AB91 was provided by the Sweet Pepper Research Group of the Institute of Cash Crops, Hebei Academy of Agriculture and Forestry Sciences. Its sterility trait is controlled by a pair of recessive nuclear genes and is genetically stable.

### Establishment of the near-isogenic line of sweet pepper genic male sterility AB91

To reduce genetic background interference, precisely localize the target gene to specific chromosomal regions, improve accuracy of gene mapping, a near-isogenic line of genic male sterile sweet pepper AB91 was developed. Fertile plants (MSms) from the sweet pepper male sterile dual-purpose line AB91 were self-crossed to generate an F2 population that segregated into recessive sterile plants (msms) and fertile plants (MSms, MSMS). To determine the genotype of the fertile plants, individual seeds were produced by self-crossing each fertile plant (MSms, MSMS) separately. During this process, anthers were collected from each F2 individual plant and stored at -80°C. The genotype of the pure fertile plants in the F2 generation was subsequently identified based on the fertility segregation observed in the F3 generation.A gene pool was constructed using the sterile plants (msms) and homozygous pure fertile plants (MSMS), thereby completing the establishment of the research population for the near-isogenic line of genic male sterile sweet pepper AB91.

### Detection of flower bud sizes at different stages

Previous cytological studies have demonstrated that the sterility in these occurs plants subsequent to the formation of microspore tetrads (Shuangxia et al., 2006). To examine the changes in gene expression levels before and after the tetrad stage, three distinct developmental stages were defined: After the formation of tetrads of microspores stage, Tetrad stage of microspores stage, and Before the formation of microspore tetrads stage (**Table 1**).

**Table 1:** Identification numbers of fertile and sterile plants at different stages of pepper genic male sterility AB91.

Fertility	After the formation of tetrads of microspores (stage 1)	Tetrad stage of microspores (stage 2)	Before the formation of microspore tetrads (stage 3)
Fertile	k1(k11, k12, k13)	k2(k21, k22, k23)	k3(k31, k32, k33)
Sterile	b1(b11, b12, b13)	b2(b21, b22, b23)	b3(b31, b32, b33)

During the flowering peak, small inflorescences were collected between 9:00 and 11:00 AM and fixed with Carnoy’s fixative for 48 hours. Flower buds of different lengths were categorized by size and stored in a 4 °C refrigerator. Anthers of different sizes were placed on a slide, and conventional slide preparation was used. The stages of the flower buds were observed and photographed under a microscope after staining with hematoxylin.

### Sample collection in different developmental stages

During the full bloom stage, to avoid errors in sequencing results caused by the corolla, style, and other structures, we selected anthers from three different developmental stages (before the tetrad stage of microspores, at the tetrad stage of microspores, and after the tetrad stage of microspores) of fertile plants with dominant homozygous alleles and sterile plants with recessive alleles from the F2 generation of the sweet pepper recessive genic male-sterile AB91 dual-purpose line for transcriptome sequencing analysis. Each sample was designed with three biological replicates.

### RNA extraction and sample quality assessment

Flower buds from various developmental stages were collected, and their anthers were meticulously dissected and placed into 2-mL centrifuge tubes. These tubes were immediately immersed in liquid nitrogen and stored at -80°C. The concentration and purity of the extracted total RNA were measured using a NanoDrop 2000 spectrophotometer (Thermo Fisher Scientific, USA). RNA integrity was assessed using an RNA Nano 6000 Assay Kit on the Agilent Bioanalyzer 2100 system (Agilent Technologies, USA). Only high-quality RNA samples (OD 260/280 = 1.8-2.2, OD 260/230≥2.0, and RIN≥7.0) were used for the construction of sequencing libraries.

### RNA library construction and library quality control

Upon successful sample quality assessment, library construction is initiated. Following completion of the library construction, the library concentration and insert size are evaluated using Qubit 2.0 and Agilent 2100, respectively. The effective concentration of the library is precisely quantified via the Q-PCR method to ensure optimal library quality. Once the library has been validated through quality inspection, high-throughput sequencing is conducted on the Illumina HiSeq 4000 platform, utilizing a paired-end sequencing read length of 150 base pairs (PE150). Yielding high-quality clean data.

### Bioinformatics analysis

The raw sequencing data are subsequently processed through data filtering to eliminate adapter sequences and low-quality reads. Clean reads were aligned, they were aligned with the reference genome(ftp://public.genomics.org.cn/BGI//pepper/Capsicum.annuum.L_Zunla-1_Release_2.0.fasta.gz) to determine their positions on the reference genome or genes, as well as to identify unique sequence features of the sequencing samples. The expression levels of transcripts and genes were quantified based on the positional information of the Mapped Reads on the genes. The Fragments Per Kilobase of transcript per Million fragments mapped (FPKM) (Trapnell et al., 2010) was employed as the metric for measuring the expression levels of transcripts or genes. Differential expression analysis between sample groups was performed using the DESeq software (Anders and Huber, 2010), with a screening criterion of Fold Change≥2 and False Discovery Rate (FDR)<0.01 to identify differentially expressed genes. Additionally, functional annotation of the differentially expressed genes was carried out using the KEGG(Kyoto Encyclopedia of Genes and Genomes) (Kanehisa et al., 2004) metabolic pathway database.

### Construction of co-expression network and selection of core genes in specific modules

The WGCNA package was employed to calculate the soft threshold using the pick Soft Threshold function and to estimate the optimal power value using the power Estimate function. A value of β = 9 was selected to transform the original matrix into an adjacency matrix, with the scale-free network fitting index R²>0.80. To further enhance the correlation of expression traits between genes, the adjacency matrix was converted into a topological overlap matrix (TOM). Gene clustering and module division were then performed using a dynamic tree-cutting criterion. Finally, the scale-free gene co-expression network was constructed using the blockwise Modules function with default parameters (Roumeliotis et al., 2012), and modules with a similarity greater than 0.8 were merged.

### Selection of core genes in specific modules and construction of interaction networks

The module membership (MM) value reflects the correlation between a gene and a module. Genes with high MM values, indicating a strong correlation with the module and high connectivity within the module, were selected as candidate genes with MM > 0.90. Cytoscape was used to display the core gene interaction network.

### qRT-PCR validation of differentially expressed genes

To assess the authenticity of the Illumina analysis and further investigate the expression patterns of DEGs, fluorescence quantitative PCR experiments were conducted using specific primers to quantify the expression of differential genes identified in the transcriptome. Elongation Factor-1α was used as a reference gene to analyze their expression patterns in sterile and fertile plants at three different developmental stages of flower buds, with three replicates for each sample. Gene names and qRT-PCR primer information are detailed in **Supplementary Table S1**.

## Results

### Morphological observations and comparison of floral organs

**Figure 1:**
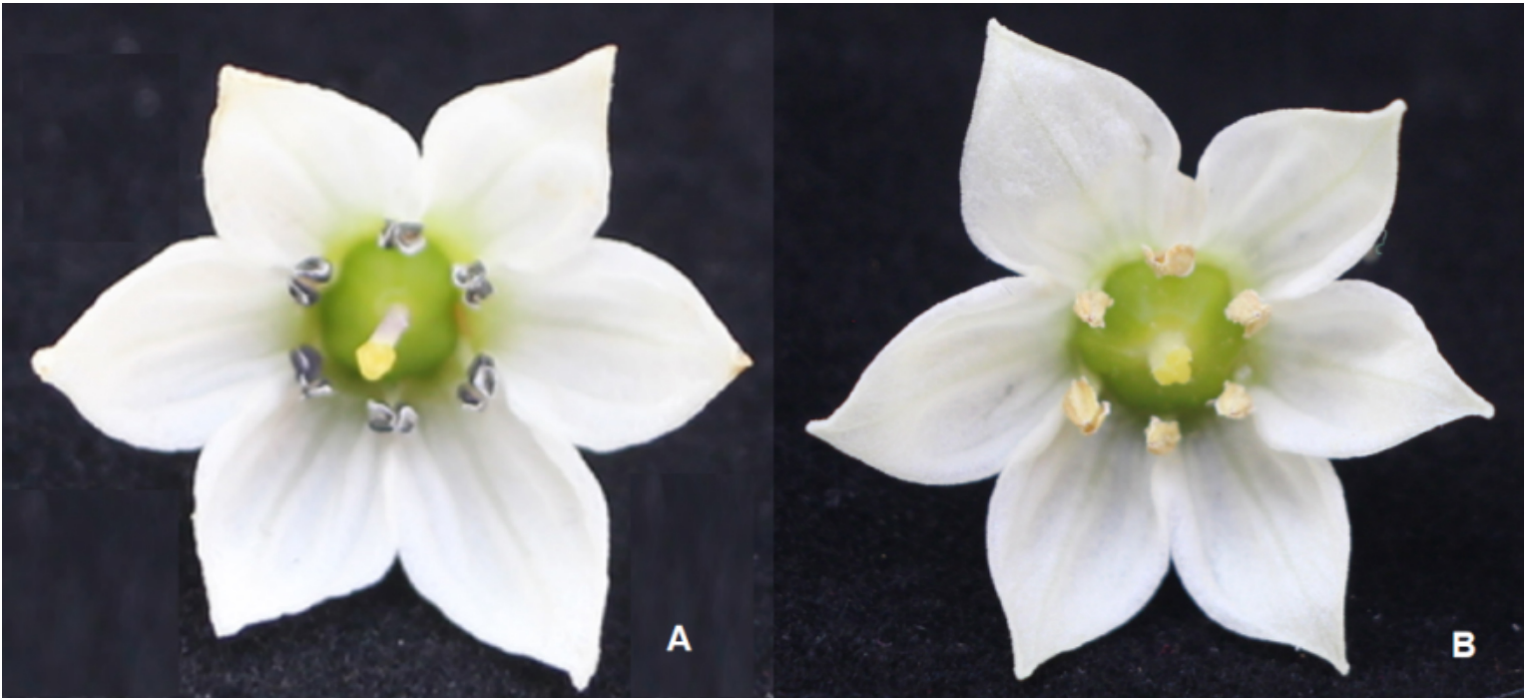
Morphological identification of sterile and fertile flowers in the pepper AB91 nuclear male sterile line. **(A)** Sterile pepper flower; **(B)** Fertile pepper flower.

There were minimal differences in the size of floral organs, flower opening degree, and petal size between fertile and sterile plants in the pepper nuclear male sterile line. The main difference was that the sterile plants had stigmas protruding about half the height of the anthers after flowering, with thin and shriveled anthers in a light purple color, and no pollen visible after dehiscence. The stigmas developed normally. In contrast, fertile plants had stigmas at or below the level of the anthers after flowering, with yellow, plump, and full anthers, and pollen covering the anthers after dehiscence (**Figure 1**).

### Relationship between flower bud morphology and three different developmental stages

**Figure 3:**
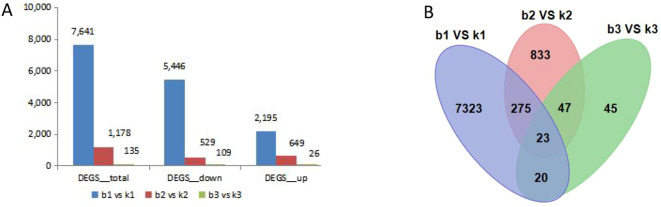
Correlation heat map and analysis of DEGs between the fertile line and male sterile line in three different stages of flower buds. **(A)** The total number of up-regulated and down-regulated DEGs. **(B)** Venn diagram of all DEGs.

**Figure 4:**
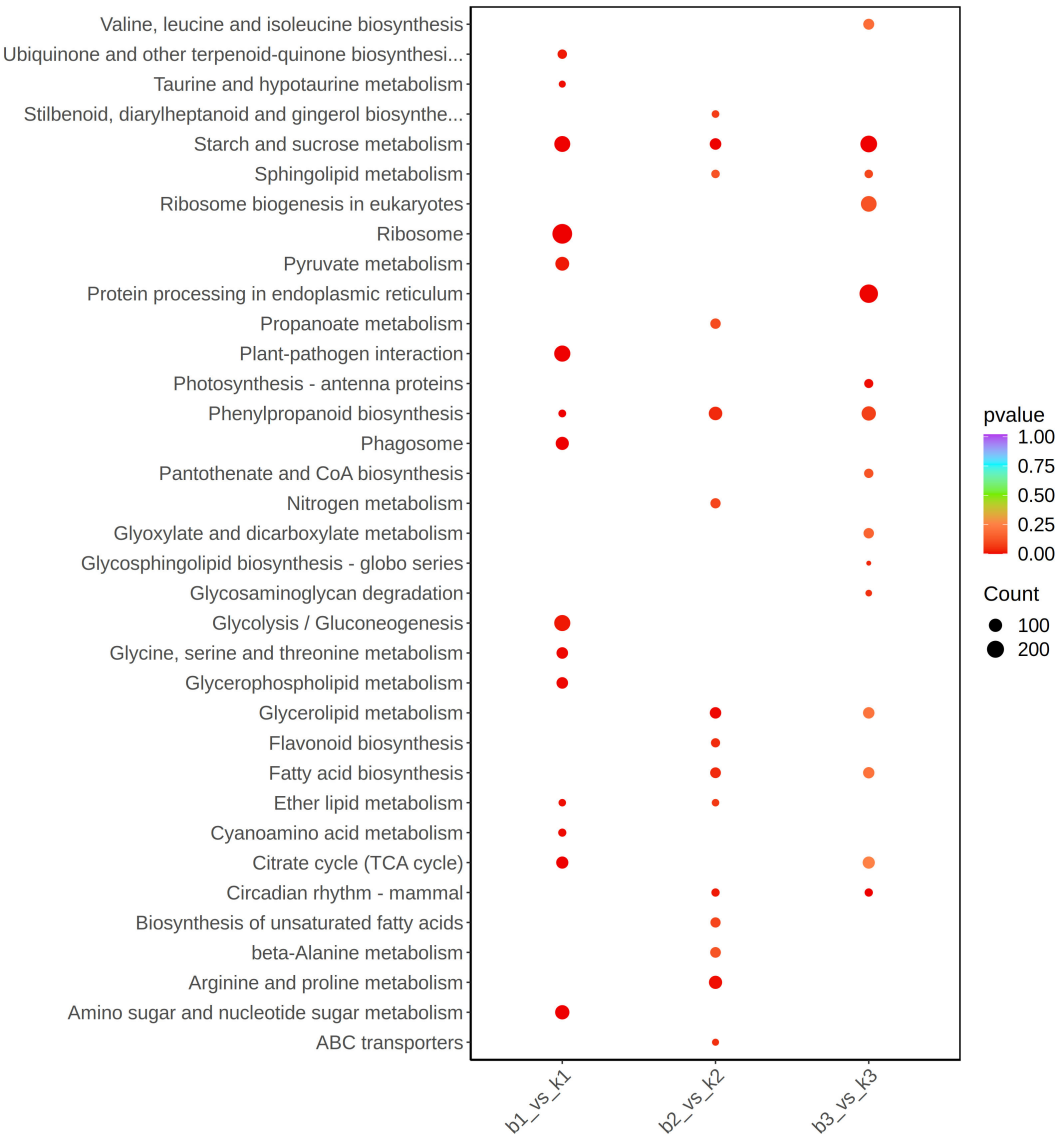
KEGG pathway enrichment scatter plot of differentially expressed genes between fertile and sterile plants in the sweet pepper genic male sterile dual-purpose line across three stages.

**Figure 6:**
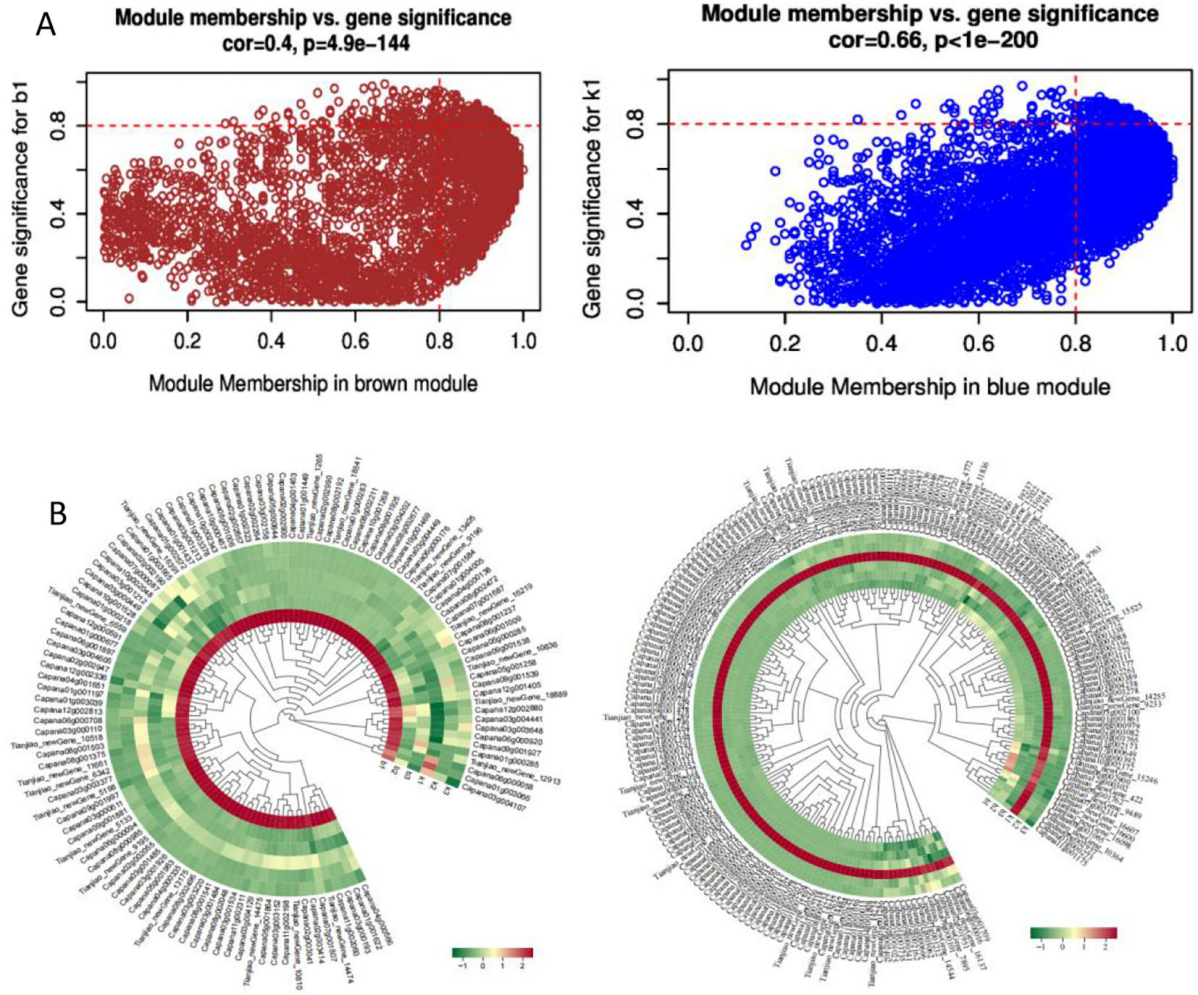
Characterization of Differentially Expressed Genes in the Brown and Blue Modules. **(A)** Scatter plot of GS-MM for the two modules; **(B)** Heatmap of expression levels of differentially expressed genes in the two modules.

Microscopic observations were performed to determine the meiotic stages of flower buds of varying sizes. The external morphology of flower buds is related to the early stages of tetrad, tetrad, and pollen maturity. The ratio of petal length to sepal length was used as a basic indicator to judge the stage of microspore development. After meiosis observation, it was found that when the sepal was higher than the petal,it was the stage before the formation of microspore tetrads. (**Figures 2A-1~3; B-1~2**); when the petal and sepal were of equal length, it was the tetrad stage (**Figures 2A-4 ~5; B-3**); and when the petal was higher than the sepal, it was the stage after the formation of microspore tetrads. (**Figures 2A-6; 2B-4**). These results provided a basis for selecting flower buds from three distinct developmental stages for subsequent transcriptome sequencing and material extraction.

**BFigure 2:**
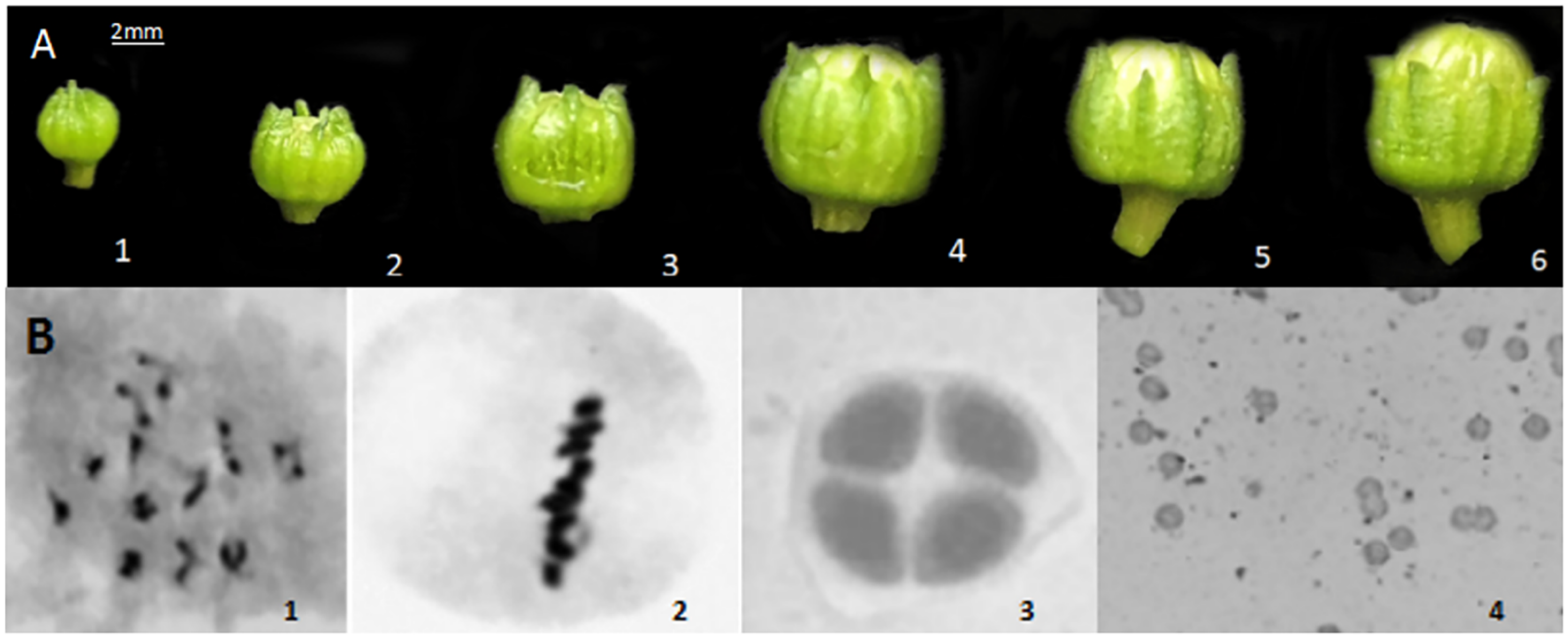
External morphology of different stages of flower buds and cytological observations. **(A)** External morphology of different stages of flower buds; **(B)** Cytological observations at different stages.

### Establishment of the research population for genic male sterility AB91 in sweet pepper

The F2 population obtained by self-crossing the fertile plants (MSms) of the sweet pepper genic male sterile dual-purpose line AB91, grown in the field, comprised 222 plants. During the flowering period, the fertility of each plant was evaluated. The results indicated that among the F2 population, there were 167 fertile plants and 55 sterile plants, with the segregation ratio of fertile to sterile plants closely matching the theoretical 3:1 ratio. Fertile plants (MSms and MSMS) from the F2 population were individually selected for seed production and then planted separately, with each single-plant population consisting of 40–50 plants. Based on the fertility segregation within each plant line, fertile plants carrying the heterozygous genic male sterile gene (MSms) and those with homozygous dominant fertility (MSMS) were identified. A total of 53 homozygous dominant fertile plants were selected from the F2 generation. Subsequently, anthers from three different developmental stages were collected from both homozygous fertile and sterile plants, based on the morphology of the flower buds, and RNA was extracted.

### Transcriptome sequencing data results

A total of 110.21Gb of Clean Data was obtained from the transcriptome sequencing, with each sample reaching 5.21Gb, and the GC ratio ranging from 42.47% to 43.73%. The percentage of Q30 was>90.71% (**Supplementary Table S2**). The alignment efficiency of the transcriptome sequencing Reads with the reference genome varied from 87.82% to 95.12%, and the sequencing data met the requirements for subsequent analysis (**Table 2**).

**Table 2:** Statistics of clean data alignment results with the reference genome.

Samples	Total reads	Mapped reads	Mapped ratio	Uniq mapped reads	Uniq mapped ratio
b1-1	35,690,100	33,111,226	92.77%	28,712,154	80.45%
b1-2	40,905,204	37,344,640	91.30%	32,372,965	79.14%
b1-3	42,055,620	39,105,594	92.99%	34,151,978	81.21%
b2-1	42,204,564	38,995,400	92.40%	33,326,345	78.96%
b2-2	44,426,558	41,113,394	92.54%	35,793,410	80.57%
b2-3	38,793,226	35,549,962	91.64%	30,897,044	79.65%
b3-1	42,113,380	38,557,802	91.56%	32,887,215	78.09%
b3-2	34,741,280	32,039,172	92.22%	27,381,961	78.82%
b3-3	44,029,360	38,667,414	87.82%	32,747,794	74.38%
k1-1	35,379,012	33,623,028	95.04%	28,414,393	80.31%
k1-2	36,910,158	34,933,040	94.64%	29,440,998	79.76%
k1-3	41,395,884	39,375,556	95.12%	33,302,013	80.45%
k2-1	50,372,154	47,846,458	94.99%	41,678,769	82.74%
k2-2	38,414,060	36,314,152	94.53%	31,531,397	82.08%
k2-3	38,324,528	35,773,744	93.34%	30,646,345	79.97%
k3-1	42,582,918	38,690,560	90.86%	33,672,541	79.08%
k3-2	46,542,606	43,993,072	94.52%	38,476,056	82.67%
k3-3	39,823,288	36,648,798	92.03%	33,150,447	83.24%

### Analysis of differentially expressed genes in different developmental stages of microspores

We compared the differentially expressed genes (DEGs) in different developmental stages of microspores. In total, 8954 DEGs were identified, which were differentially expressed at different development stages. The number of differentially expressed genes had very high variance among different development stages. The largest number of DEGs was found between b1 and k1, where 7641 DEGs were identified. The libraries of b2 vs. k2 and b3 vs. k3 had 5446 and 2195 DEGs, respectively. Among those DEGs, the three libraries had 23 common DEGs. There were 2195 up-regulated DEGs in b1 vs. k1, 649 up-regulated DEGs in b2 vs. k2, and 26 up-regulated DEGs in b3 vs. k3 (**Figure 3**). This suggests that genotype-specific DEGs may contribute to phenotypic differences.

### KEGG enrichment analysis of DEGs

The KEGG enrichment Analysis was used to identify significantly changed pathways in the AB91 recessive nuclear male sterile material and fertile material. A total of 112 metabolic pathways were enriched across the three periods. **Figure 4** shows the top 15 enriched metabolic pathways for the three periods. The first period and the second period were significantly enriched in “Ether lipid metabolism (ko00565)”; the second period and the third period were significantly enriched in “Sphingolipid metabolism(ko00600)”, “Glycerolipid metabolism(ko00561)”,”Fatty acid biosynthesis(ko00061)”, and “Circadian rhythm-mammal(ko04710)”; the first period and the third period were significantly enriched in “Citrate cycle(ko00020)”. The three periods commonly enriched metabolic pathways were “Starch and sucrose metabolism(ko00500)” and “Phenylpropanoid biosynthesis(ko00940)”. From **Table 3**, it can be seen that genes in four metabolic pathways showed significant changes during the three stages of pollen development, namely “Fatty acid biosynthesis (ko00061)”, “Starch and sucrose metabolism (ko00500)”, “Citrate cycle (ko00020)”, and “Phenylpropanoid biosynthesis (ko00940)”.

**Table 3:** Statistics of the number of differentially expressed genes in eight KEGG enrichment pathways of male sterility in bell pepper nucleus at three periods.

KEGG	Stage 1		Stage 2		Stage 3	
	No.up DEGs	No.down DEGs	No.up DEGs	No.down DEGs	No.up DEGs	No.down DEGs
ko00020	1	22	3	4	3	3
ko00061	0	11	3	6	1	5
ko00500	1	34	2	6	3	6
ko00561	1	5	2	4	0	5
ko00565	1	7	0	6	0	0
ko00600	1	5	5	0	4	0
ko00940	9	3	3	3	5	2
ko04710	0	5	0	4	0	4
total	14	92	18	33	16	25

### Co-expression network analysis of genes in different genotypes with WGCNA

In the present study, co-expression networks were built on the basis of pairwise correlations among genes according to the trends of gene expression in all examined samples. The endogram showed that 9 unique modules were identified, with each module depicted by a different colored branch, and each gene depicted by a leaf. The heatmap in **Figure 5A** illustrates the similarity among genes and between modules. The modules are categorized into three distinct clusters: one cluster includes most of the Brown module, a portion of the blue module, and the Turquoise module; another cluster consists primarily of the blue module; and the remaining modules form the third cluster.

**BAFigure 5:**
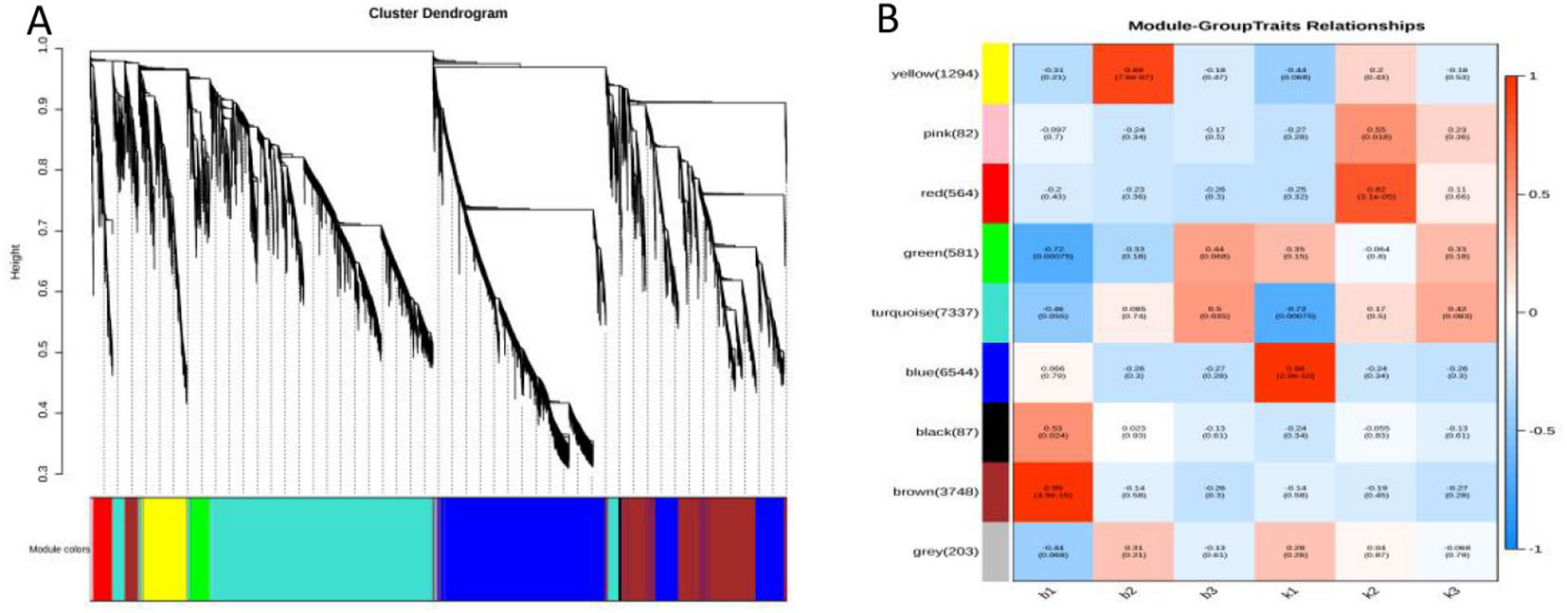
Result of gene co-expression network construction. **(A)** Hierarchical cluster tree showing co-expression modules identified by WGCNA. **(B)** Module–tissue association. Each row corresponds to a module.

The expression levels of genes within the nine co-expression modules were correlated with the fertility and sterility of plants at three distinct time points, as illustrated in **Figure 5B**. Each cell in the figure comprises the corresponding correlation coefficient and P-value, with positive correlations indicated in red and negative correlations in blue. Notably, the gene expression levels in the brown module (r = 0.99, P = 4.9e-15) and the blue module (r = 0.96, P = 2.9e-10) showed extremely strong correlations with the period after the formation of four-tentacled microspores in sterile and fertile plants (**Supplementary Tables S3**, **S4**). Therefore,the brown module and the blue module were selected as the key modules for further analysis in this study.

### Expression characteristics of key modules

The module membership (MM) value indicates the correlation between a gene and its respective module. As illustrated in **Figure 6, a** total of 320 differentially expressed genes with MM ≥ 0.8 were identified within the Blue and Brown modules. Specifically, the Brown module included 118 differentially expressed genes whose expression levels peaked in the first period of the sterile plants after the formation of tetrad microspores (**Supplementary Table S5**). Conversely,the Blue module contained 202 differentially expressed genes that exhibited high expression levels in the first period of the fertile plants (**Supplementary Table S6**). Notably, the timing of expression for these differentially expressed genes aligns closely with the cytological observations (Shuangxia et al., 2006), all of which are concentrated after the formation of tetrad microspores. This alignment provides crucial evidence for further exploring the relationship between gene expression regulation and cellular developmental processes.

### Screening of core genes associated with sterility in the two specific modules of sweet pepper

Among the blue and brown modules, there are 320 differentially expressed genes with MM≥ 0.8.The functions of these genes were predicted by comparing and annotating them with their homologous genes. The results revealed that six of these genes were enriched in five metabolic pathways: Sphingolipid metabolism (ko00600), Starch and sucrose metabolism (ko00500), Glycerolipid metabolism (ko00561), Ether lipid metabolism (ko00565), and Phenylpropanoid biosynthesis (ko00940). These six genes Capana09g000101, Capana03g004068, Capana01g001338, Capana10g002261, Capana06g001592, and Capana02g002990 can be considered as core genes associated with genic male sterility(**Table 4**). Notably, Capana10g002261 was enriched in two metabolic pathways (ko00600 and ko00561), suggesting that it may be located at the intersection of interrelated pathways.

**Table 4:** Core genes related to pepper genic male sterility and their functional annotations.

Gene	KEGG	Moudle	Annotation
Capana09g000101	ko00500	blue	PREDICTED: Capsicum annuum UDP-glucuronate 4-epimerase 1 (LOC107840845), mRNA
Capana03g004068	ko00600	blue	PREDICTED: Capsicum annuum alkaline ceramidase 3-like (LOC107864315), transcript variant X2, mRNA
Capana10g002261	ko00565/ko00561	blue	PREDICTED: Capsicum annuum triacylglycerol lipase SDP1 (LOC107843863), mRNA
Capana01g001338	ko00561	blue	PREDICTED: Capsicum annuum lysophospholipid acyltransferase LPEAT2 (LOC107872567), transcript variant X2, mRNA
Capana06g001592	ko00940	blue	PREDICTED: Capsicum annuum peroxidase 41-like (LOC107874883), mRNA
Capana02g002990	ko00940	brown	PREDICTED: Capsicum annuum peroxidase 51 (LOC107860206), mRNA

### Construction of the interaction network of core genes in specific modules related to nuclear male sterility in pepper

**Figure 7:**
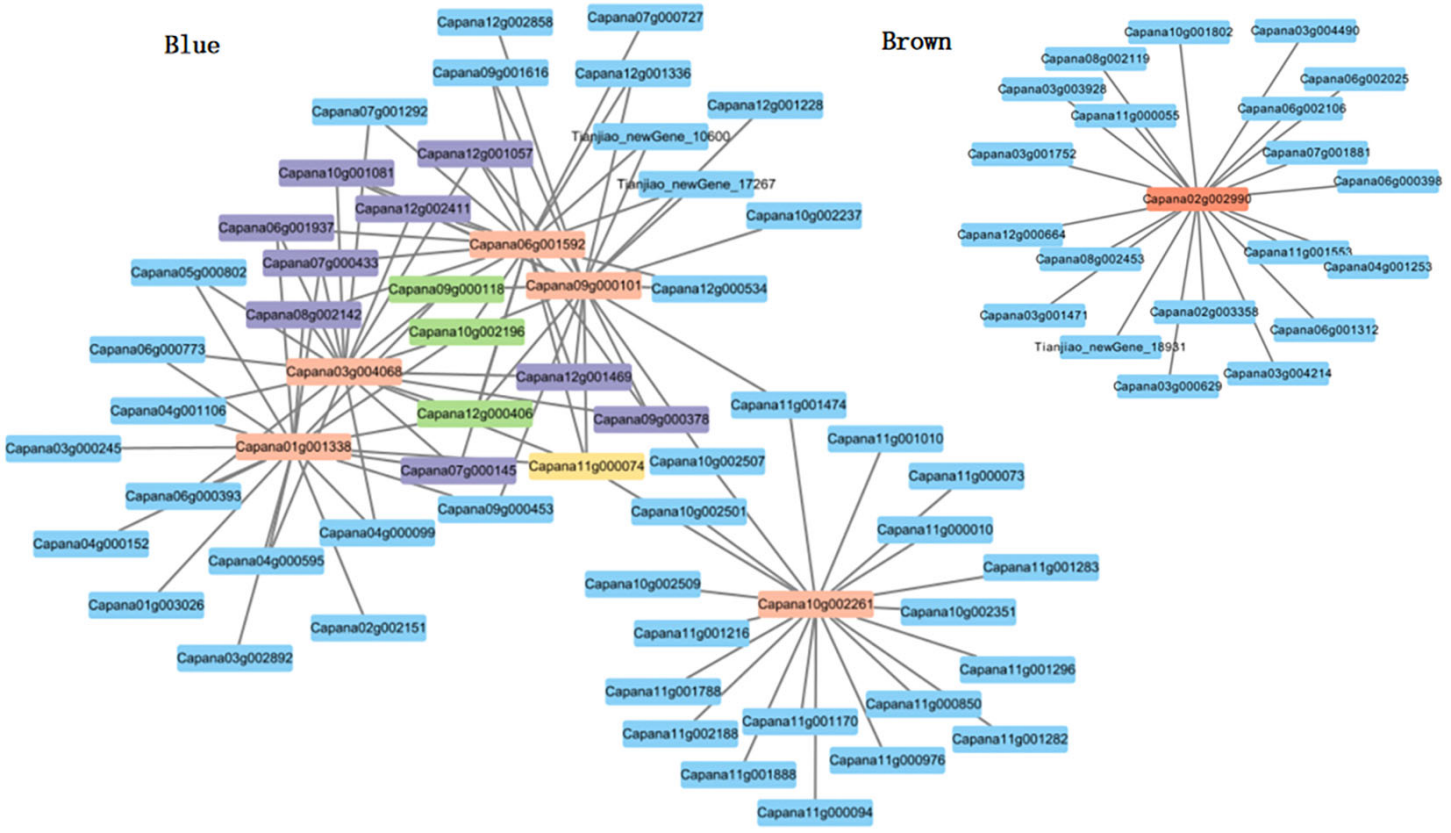
Gene co-expression network and core genes in the blue and brown modules.

Utilizing cytoscape for construction and visualization, we delineated the interaction network of the nine core genes and their top 20 associated genes based on weight values (**Figure 7**). Within this network,we identified one gene (Capana11g000074) that interacts with 5 core genes.There are 3 genes (Capana12g000406, Capana09g000118, Capana10g002196) interacting with 4 core genes.Nine genes (Capana12g001469, Capana09g000378, Capana07g000145, Capana18g002142, Capana07g000433, Capana06g001937, Capana10g001081, Capana12g001057, Capana12g002411) were found to interact with 3 core genes. These 13 genes exhibit high connectivity in the co-expression network, and it is hypothesized that they may be associated with the process of pollen inactivation in sweet pepper genic male sterility.

### Quantitative validation of core genes

**Figure 8:**
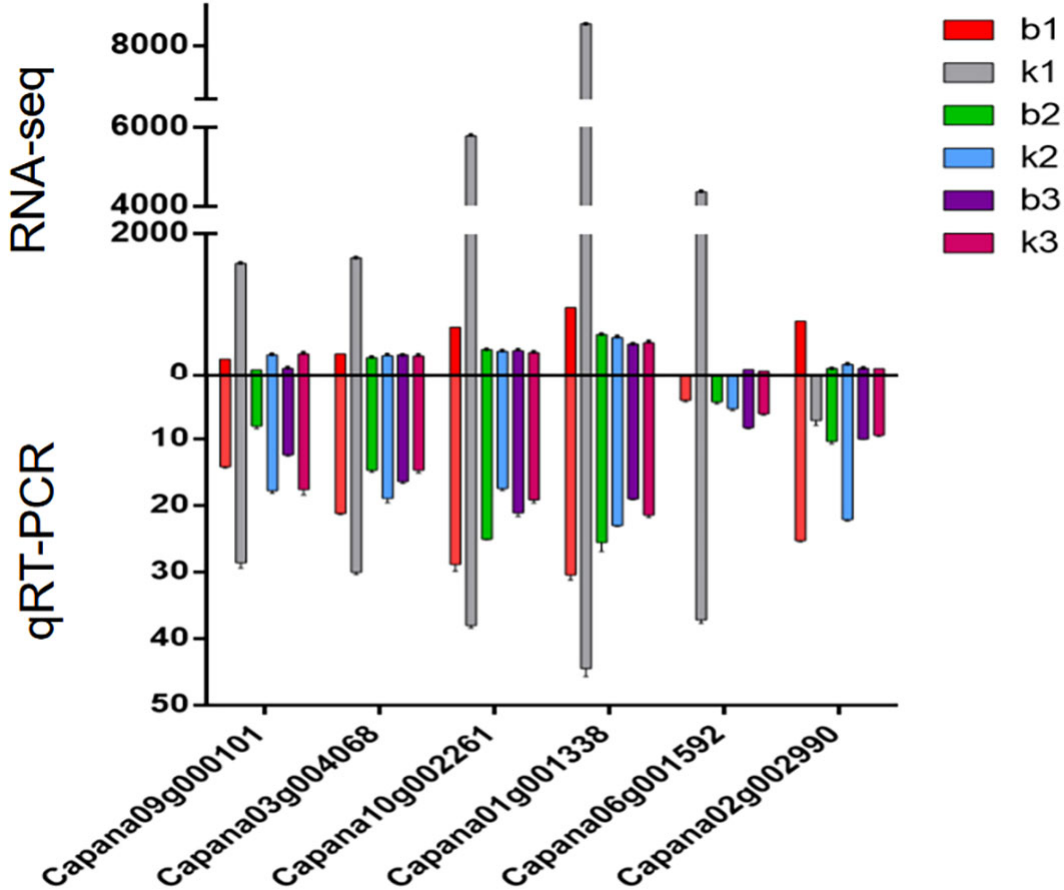
Expression analysis of the six core genes associated with genic male sterility in sweet pepper.

We selected core genes from the Blue and Brown modules for analysis. A systematic examination of gene expression levels across three distinct developmental stages showed that, in the second and third stages, gene expression levels did not significantly differ between sterile and fertile plants. However, in the first stage, the expression levels of the six core genes were significantly lower in sterile plants than in fertile plants. After the formation of tetrad microspores (the first stage) is a critical point for changes in gene expression. Abnormal regulation of gene expression during this period may play a crucial role in determining plant fertility (**Figure 8**).

## Discussion

Harnessing the male sterility trait in plants to develop male sterile lines is a vital approach for simplifying seed production processes and reducing costs in the utilization of heterosis in crops. Pollen abortion represents the phenotypic manifestation of male sterility in plants. Elucidating the complete process and molecular mechanisms of pollen development is both fundamental and critical for studying male sterility. Pollen development involves the expression regulation of a multitude of genes. Transcriptomic analysis of pollen development mutants is one of the key methods for obtaining dynamic gene expression profiles in pollen. This approach can identify a large number of pollen development-related genes, which aids in understanding the characteristics and molecular regulatory mechanisms of pollen development at a holistic level (Guo et al., 2016).

### ko00500 and ko00940 are key metabolic pathways regulating genic male sterility in sweet pepper AB91

The integration of transcriptome data with the Weighted Gene Co-expression Network Analysis (WGCNA) algorithm has proven to be a powerful approach for identifying core genes involved in plant growth and development. This method has been widely utilized in studies focusing on the morphogenesis and regulatory mechanisms of plant organs, including flowers, leaves, and fruits, as well as in predicting the functions of previously uncharacterized genes (Langfelder and Horvath, 2000; Hollender et al., 2014; Luo et al., 2019). In the KEGG analysis, differentially expressed genes (DEGs) were predominantly enriched in four key pathways: phenylpropanoid biosynthesis, starch and sucrose metabolism, lipid metabolism, and the tricarboxylic acid cycle. This enrichment suggested that these metabolic pathways are crucial in driving the observed differences between groups and are likely central to regulating phenotypic changes. Meanwhile, the WGCNA analysis highlighted pathways such as Sphingolipid metabolism (ko00600), Starch and sucrose metabolism (ko00500), Glycerolipid metabolism (ko00561), Ether lipid metabolism (ko00565), and Phenylpropanoid biosynthesis (ko00940). The consistent enrichment of Starch and sucrose metabolism and Phenylpropanoid biosynthesis in both analyses indicates that these pathways may play significant roles in the biological process of pollen abortion in sweet pepper AB91, likely regulated by multiple factors. Pollen development and maturation demand significant energy resources. Starch and sucrose serve as critical energy storage compounds within plant cells (Wei et al., 2013; Wu et al., 2013; Sang-Kyu et al., 2022). In this study, the down regulation of most genes involved in starch and sucrose metabolism and synthesis pathways resulted in a shortage of available energy substrates for pollen cells. This energy deficit can impair normal pollen development, leading to abortion due to insufficient energy during the developmental process. Lignin, a key end product of the phenylpropanoid biosynthesis pathway,is essential for anther development (Mishima et al., 2018; Zhang et al., 2020). In this study, the upregulation of lignin synthesis-related genes may cause uneven distribution or excessive accumulation of lignin within the anthers. This can disrupt the anther Microenvironment and negatively affect pollen development and function, ultimately leading tomale sterility.

### After the formation of tetrad microspores is a critical juncture for changes in gene expression related to genic male sterility in sweet pepper AB91

Regulating plant fertility is a complex process that involves multiple genes and the coordinated actions of various pathways (Yang and Lin, 2018). The 202 differentially expressed genes in the Blue module show high expression levels in the first period of fertile plants (after the formation of tetrad microspores), but these genes are significantly down regulated or even silenced in sterile plants. The normal high expression of blue module genes is crucial for maintaining plant fertility, and their absence or abnormal expression is likely a key factor leading to the sterile phenotype. In sterile plants, the down regulation of blue module genes disrupts thesynthesis of key substances required for microspore development and the regulation of the cell cycle, preventing microspores from developing into mature, fertile pollen grains (Zhang et al., 2020). In contrast, the expression of Brown module genes is abnormally upregulated in the first period of sterile plants, suggesting that they may interfere with normal fertility regulation pathways and drive the plant towards sterility. For instance, the overexpression of these genes may disrupt the balance of material transport between the tapetum and microspores, leading to developmental defects in microspores (Yang et al., 2025). The differences in expression patterns between these two modules reveal the synergistic and antagonistic relationships of gene functions in the regulation of plant fertility.

In this study, we identified the abnormal expression of six core genes following the formation of tetrad microspores. Five of these key genes, which belong to the Blue module, were found to be down regulated in sterile plants. These genes may contribute to plant infertility by regulating microspore development, tapetal function, or hormone balance. Similar findings have been reported in model plants such as rice (Abbas et al., 2021) and Arabidopsis (Ito et al., 2007), where abnormal expression of specific genes during early microspore development has been closely linked to male sterility. These previous studies further underscore the critical role of the post-tetrad microspore formation period in regulating plant fertility.

### Identification of six core genes associated with genic male sterility in sweet pepper AB91

Male sterility is a critical area of research in plant reproductive development, with its molecular mechanisms encompassing a variety of biological processes, including energy metabolism, reactive oxygen species balance, pollen wall formation, hormone signaling, and programmed cell death (PCD) (Chen et al., 2020; Zhao et al., 2021; Wang et al., 2022). In this study, we identified six core genes associated with genic male sterility in sweet pepper AB91, using transcriptome data from fertile and sterile plants at three distinct developmental stages. Sequence alignment revealed that these six genes encode UDP-glucuronate 4-epimerase 1, alkaline ceramidase 3-like, triacylglycerol lipase SDP1, lysophospholipid acyltransferase, peroxidase 41-like, and peroxidase 51.These six enzymes involved in cell wall synthesis, signal transduction, energy supply, membrane structure maintenance, and redox balance form a tightly interconnected regulatory network. Any abnormality in one link could disrupt the balance of male reproductive development, potentially leading to male sterility.Additionally, 13 genes were found to interact with multiple core genes. Abnormalities in any of these genes can disrupt the balance of male reproductive development, leading to male sterility.The functions of these six core genes in sweet pepper genic male sterility have not been previously reported and warrant further investigation.

These findings provide a theoretical foundation for understanding the mechanisms underlying genic male sterility in sweet pepper and pave the way for the utilization of heterosis, expansion of fertility gene resources, and advancement of hybrid breeding in sweet pepper.

## Data Availability

The data presented in the study are deposited in the GenBank repository, accession number PRJNA1190054.
